# Purifying selection drives distinctive arsenic metabolism pathways in prokaryotic and eukaryotic microbes

**DOI:** 10.1093/ismeco/ycae106

**Published:** 2024-08-20

**Authors:** Lijuan Li, Songcan Chen, Ximei Xue, Jieyin Chen, Jian Tian, Lijuan Huo, Tuo Zhang, Xibai Zeng, Shiming Su

**Affiliations:** Institute of Environment and Sustainable Development in Agriculture, Chinese Academy of Agricultural Sciences/Key Laboratory of Agricultural Environment, MARA, Beijing 100081, P.R. China; Division of Microbial Ecology, Center for Microbiology and Environmental Systems Science, University of Vienna, Vienna 1030, Austria; Institute of Urban Environment, Key Laboratory of Urban Environment and Health, Chinese Academy of Sciences, Xiamen 361021, P.R. China; Institute of Plant Protection, State Key Laboratory for Biology of Plant Diseases and Insect Pests, Chinese Academy of Agricultural Sciences, Beijing 100193, P.R. China; Biotechnology Research Institute, Chinese Academy of Agricultural Sciences, Beijing 100081, P.R. China; School of Environment and Resources, Taiyuan University of Science and Technology, Taiyuan 030024, P.R. China; School of Environmental and Life Science, Nanning Normal University, Nanning 530100, P.R. China; Institute of Environment and Sustainable Development in Agriculture, Chinese Academy of Agricultural Sciences/Key Laboratory of Agricultural Environment, MARA, Beijing 100081, P.R. China; Institute of Environment and Sustainable Development in Agriculture, Chinese Academy of Agricultural Sciences/Key Laboratory of Agricultural Environment, MARA, Beijing 100081, P.R. China

**Keywords:** arsenic biotransformation genes (ABGs), arsenic detoxification, eukaryotic and prokaryotic microorganisms, gene distribution, selection pressure

## Abstract

Microbes play a crucial role in the arsenic biogeochemical cycle through specific metabolic pathways to adapt to arsenic toxicity. However, the different arsenic-detoxification strategies between prokaryotic and eukaryotic microbes are poorly understood. This hampers our comprehension of how microbe–arsenic interactions drive the arsenic cycle and the development of microbial methods for remediation. In this study, we utilized conserved protein domains from 16 arsenic biotransformation genes (ABGs) to search for homologous proteins in 670 microbial genomes. Prokaryotes exhibited a wider species distribution of arsenic reduction- and arsenic efflux-related genes than fungi, whereas arsenic oxidation-related genes were more prevalent in fungi than in prokaryotes. This was supported by significantly higher *acr3* (arsenite efflux permease) expression in bacteria (upregulated 3.72-fold) than in fungi (upregulated 1.54-fold) and higher *aoxA* (arsenite oxidase) expression in fungi (upregulated 5.11-fold) than in bacteria (upregulated 2.05-fold) under arsenite stress. The average values of nonsynonymous substitutions per nonsynonymous site to synonymous substitutions per synonymous site (dN/dS) of homologous ABGs were higher in archaea (0.098) and bacteria (0.124) than in fungi (0.051). Significant negative correlations between the dN/dS of ABGs and species distribution breadth and gene expression levels in archaea, bacteria, and fungi indicated that microbes establish the distinct strength of purifying selection for homologous ABGs. These differences contribute to the distinct arsenic metabolism pathways in prokaryotic and eukaryotic microbes. These observations facilitate a significant shift from studying individual or several ABGs to characterizing the comprehensive microbial strategies of arsenic detoxification.

## Graphical Abstract

**Graphical Abstract:**
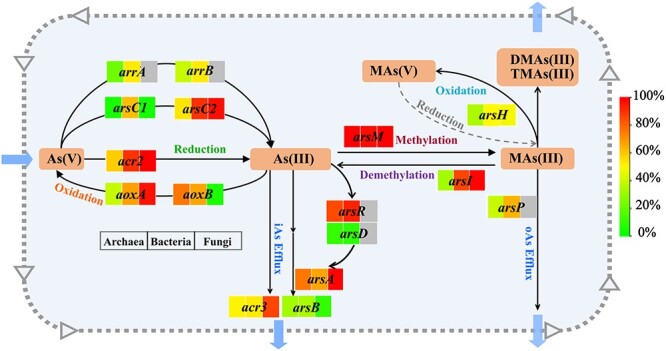

## Introduction

Microbes drive the biogeochemical cycle of arsenic through their impact on arsenic mobilisation at micro-interfaces (e.g. soil–water, soil–root, and soil–gas interfaces) [1] and across multi-phases (e.g. liquid, gas, and solid phases) [2]. Arsenic exposure poses significant challenges to understanding the origin of life [2] and current human health [3]. To cope with arsenic toxicity, microbes have developed diverse strategies, which vary depending on the microbial species and niches [4, 5]. Correspondingly, these detoxification strategies drive the adaptation and divergence of microbial arsenic metabolism pathways [6]. Some prokaryotic and eukaryotic microbes exhibit distinct arsenic resistance via the biotransformation of arsenic species [2]. However, the diverse patterns of arsenic metabolism in prokaryotes and eukaryotes, and the mechanisms by which natural selection drives these differences, remain poorly understood. This lack of knowledge hampers our comprehension of how microbe–arsenic interactions drive the arsenic cycle and the development of microbial methods for remediation.

The limited discovery of arsenic biotransformation genes (ABGs) contributes to this existing knowledge gap. Consequently, extensive research has focused on exploring functional ABGs, particularly in prokaryotic microbes. ABGs mediating eukaryotic arsenic detoxification remain largely unknown [7]. Another contributing factor is the slower progress in obtaining high-quality genome-wide sequences of fungi, compared with those of bacteria [8], hindering our understanding of ABGs in eukaryotic microbes. To date, the Department of Energy Joint Genome Institute (JGI) has released >2393 high-quality fungal genomes (Berkeley Lab, Berkeley, CA, USA). Molecular and genetic studies have identified >30 ABGs in microbes [2, 9], including the arsenite [As(III)] efflux permease genes *arsB* and *acr3* [9], methylarsenite [MAs(III)] efflux permease gene *arsP* [10], As(III) oxidase genes *aoxA* and *aoxB* [11], MAs(III) oxidase gene *arsH* [12], arsenate [As(V)] reductase gene *arsC*, resistance reduction gene *acr2* [13, 14], respiratory reduction genes *arrA* and *arrB* [15], arsenic methylation gene *arsM* [16], demethylation gene *arsI* [17], and transcriptional repressor genes *arsR* [18] and *arsD* [19]. While most of these ABGs were identified in prokaryotic genomes [20], some genes, such as *acr3* [21], *arsC2* [13], and *arsM* [22], have also been validated in eukaryotic genomes. Certain ABGs (e.g. *arsR* and *arsC*) that originated in prokaryotic microbes have homologous genes in eukaryotic genomes [23, 24]. Therefore, we believe that it is now feasible to explore the diversity patterns of arsenic metabolism pathways in prokaryotic and eukaryotic microbes.

Natural selection is a key driving force behind genomic adaptation in response to environmental changes, including arsenic exposure [25, 26]. Certain prokaryotes utilize arsenic oxyanions as an energy source through the oxidization of As(III) [27]. This could have conferred a selective advantage to early-stage prokaryotes, enabling them to cope with widespread arsenic stress [27, 28]. Functional genes involved in As(V) reduction and As(III) resistance exhibit stronger purifying selection in *Rhodanobacter* isolated from arsenic-contaminated fields than those isolated from uncontaminated land [29]. However, fungi possess unique morphological and biochemical features that set them apart from bacteria, and these confer a selective advantage [8, 30]. The microhabitat surrounding fungal hyphae in soil can create an environment conducive to horizontal gene transfer, which has considerable evolutionary implications for fungal interactions [30]. Various computational methods exist for evaluating natural selection in protein-coding sequences [31]. Among them, the ratio of nonsynonymous substitutions per nonsynonymous site (dN) to synonymous substitutions per synonymous site (dS), labelled as dN/dS, is one of the most widely used approaches for testing the strength and mode of natural selection [32], revealing the pace of amino acid-altering substitutions relative to synonymous substitutions [33]. The dN/dS of genes is thought to be strongly linked to gene expression intensity and species distribution breadth in microbes [34, 35]. This, in turn, determines the functional role of genes in microbial niches [32].

Therefore, we hypothesized that different niches with varying levels of arsenic exposure exert strong selective pressure on prokaryotic and eukaryotic microbes, leading to the development of diverse mechanisms by which they resist arsenic and adapt their metabolism. Here, the distribution and co-occurrence relationships of 16 homologous ABGs across 670 microbial genomes (archaea, bacteria, and fungi) were investigated. The distinct expression of key ABGs between prokaryotic and eukaryotic microbes was also validated after exposure to arsenic. The strength and mode of natural selection of ABGs in microbes were further explored based on the dN/dS. The species distribution breadth, expression level, and relationships with adaptive evolution were finally established for ABGs.

## Materials and methods

### Selection of genomes and phylogenetic tree construction

In total, 670 completely sequenced genomes were selected, including 487 bacterial genomes (representing 36 phyla, 110 classes, 311 orders, 487 families, and 487 genera), 41 archaeal genomes (representing three phyla, 12 classes, 21 orders, 41 families, and 41 genera) from the National Center for Biotechnology Information (NCBI; FTP site, http://ftp.ncbi.nih.gov/genomes/genebank/bacteria/), and 142 fungal genomes (six phyla, 64 classes, 311 orders, 142 families, and 142 genera) from the JGI database (https://genome.jgi.doe.gov/portal/fungi/fungi.download.html) (Dataset S1). A phylogenetic tree was constructed by concatenating the proteome sequences of the 670 genomes using CVTree 3.0 software (http://cvtree.online/v3/cvtree/index.html) to examine the phylogenetic distribution of homologous ABGs [36]. The tree was visualized and edited using iTOL v6 (https://itol.embl.de/). The root tree was constructed using the *Halosimplex litoreum* genome (Archaea; Euryarchaeota, See online supplementary material for a colour version of Fig. S1).

### Reference sequences and identification of ABGs

The EggNOG v4.5 database was used to annotate reference protein sequences encoded by ABGs that were downloaded from UniProt or NCBI. The annotations included Clusters of Homologous Groups (COGs) functional categories [37], Gene Ontology (GO) terms [38], Kyoto Encyclopedia of Genes and Genomes pathways [39], and protein family (Pfam) domains [40] (Dataset S2). The reference sequences were subsequently aligned and annotated to determine the biochemical properties of ABG-encoded proteins (Table S1). The homologous protein sequences encoded by ABGs were searched against the 670 genomes using the HMMER tool (E value, 1e^−10^) with the corresponding hidden Markov model from the Pfam database. Moreover, Batch CD-Search in the NCBI Conserved Domain Database (http://www.ncbi.nlm.nih.gov/Structure/bwrpsb/bwrpsb.cgi) (identity, >30%; E-value, 1e^−10^) was used to examine the conserved domains of homologous ABGs. An ABG distribution heatmap for the 670 genomes and co-occurrence networks of ABGs in prokaryotes and eukaryotes were created by using the predicted ABG-encoded protein homologs. The ratio of the number of target genes to the total number of genomes was employed to represent the mean copy number of each ABG. The ratio of the number of species harbouring the target gene to the total number of genomes was employed to represent the distribution breadth for each ABG [41].

### Relative expression of ABGs in microbes after As(III) exposure using real-time polymerase chain reaction

Twelve strains were chosen in this study after searching the strain bank of the China General Microbiological Culture Collection Center (Institute of Microbiology, Chinese Academy of Sciences, Beijing, China) as experimental strains containing crucial transformation-associated genes (*aoxA*, *arsM*, and *acr3*). The safety, easy culturability, and phyla diversity of strains were also taken into consideration. The experimental strains included six bacteria (representing four phyla: Actinobacteria, Bacteroidetes, Firmicutes, and Proteobacteria) and six fungi (representing three phyla: Ascomycota, Basidiomycota, and Mucoromycota). Fungal strains were cultured in a reference medium (Table S2) containing 1 mM NaAsO_2_ (Sigma-Aldrich Chemical Company, St. Louis, MO, USA) at 25°C with 140 rpm shaking, and bacteria were grown at 37°C with 200 rpm of shaking. The growth of all strains in the medium with 1 mM NaAsO_2_ was satisfactory based on preliminary experiments. For fungi, harvesting was performed after 7 days (2.0 < OD_600_ < 4.0); for bacteria, harvesting was performed after 5 days (1.0 < OD_600_ < 3.0). Fungi and bacteria grown in arsenic-free media were set as the control groups. The copy number of genes 16S rRNA (reference gene for bacteria), 18S rRNA (reference gene for fungi), *aoxA*, *acr3*, and *arsM* (details provided in Supporting Information Text S1,Table S3 and Table S4) was quantified using quantitative real-time polymerase chain reaction. The 2^−ΔΔCT^ method was used to analyse the relative expression of each target gene (*aoxA*, *acr3*, and *arsM*) [42]. The log_2_ ratios were generated by comparing the copy number of each ABG in bacteria or fungi to the copy number of 16S or 18S rRNA. Mean and standard error values were calculated after computing the expression fold-change for each strain based on three biological replicates.

### Calculation of dN/dS of ABGs

The recombinase gene *recA* (Family accession: TIGR02012.1), DNA topoisomerase (ATP-hydrolyzing) subunit B gene *gyrB* (TIGR01059.1), elongation factor G gene *fusA* (TIGR00484.1), and isoleucine-tRNA ligase gene *ileS* (TIGR00392.1) are all highly conserved housekeeping genes related to RNA transcription (Table S5). The 670 genomes were searched using the protein family models for these genes, which were obtained from NCBI. A multi-step process was used to evaluate the strength and mode of natural selection of each ABG in eukaryotic and prokaryotic microbes. The methodology included sequence alignment, stop codon removal, phylogenetic analysis, and estimation of the dN/dS at the codon level. Stop codons were removed before the analysis. TranslatorX 14.0 software was used to translate coding protein sequences into protein sequences (http://www.translatorx.co.uk/). FastTree 2.1.11 software (http://www.microbesonline.org/fasttree/#Install) was used to build phylogenetic trees based on the aligned protein sequences. HyPhy 2.5.2 software was used to estimate the dN/dS in accordance with the reference instructions (https://stevenweaver.github.io/hyphy-site/tutorials/current-release-tutorial/). Subsequently, the associations among the strength of natural selection of homologous ABGs, the expression of key ABGs, and species distribution were established.

## Results and discussion

### Phylogenetic distribution of homologous ABGs in prokaryotic and eukaryotic microbes

**Figure 1:**
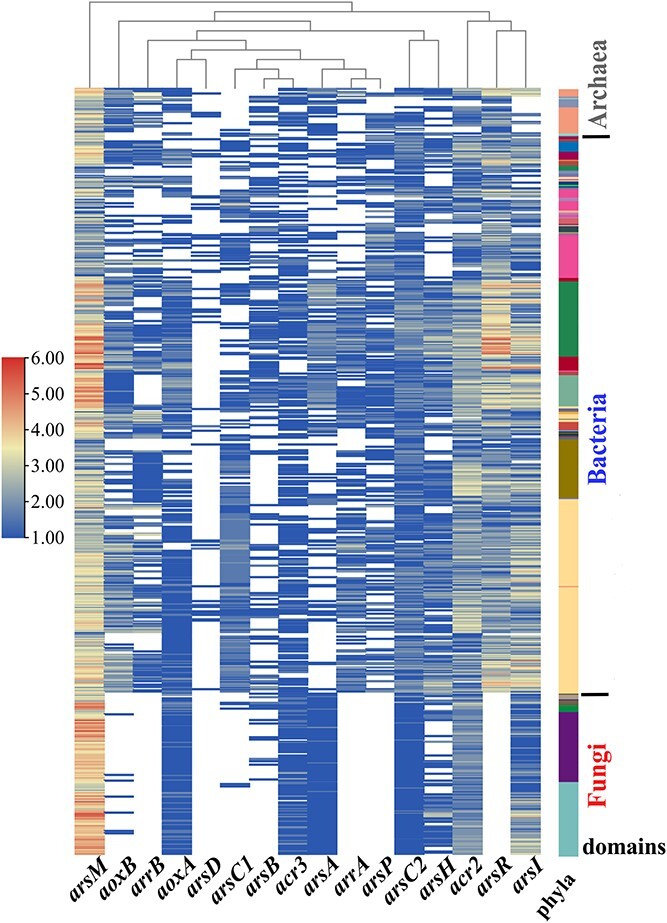
Copy numbers (normalized as log_2_) of arsenic biotransformation gene (ABG) homologs in archaea, bacteria, and fungi. The value matrix on the left is presented using a continuous colour scheme. A comprehensive list of 670 microbes is provided, showing their taxonomic affiliation at the phylum level. The phylogenetic tree was constructed based on a proteome comparative analysis of 670 microbes, shown in Fig. S1. The phylum colours from Fig. 1 match those in Fig. S1.

Homologous ABGs were widely distributed among the major lineages of archaea, bacteria, and fungi (Fig. 1 and See online supplementary material for a colour version of Fig. S1). Among the 45 phyla classified, Proteobacteria and Actinobacteria showed a relatively wider distribution for all 16 ABGs. A wider distribution of *aoxA*, *arsM*, *arsA*, *acr3*, and *arsI* in Ascomycota and of *arsD*, *arsP*, *arsC1*, and *arsC2* in Firmicutes was also observed (See online supplementary material for a colour version of Fig. S2). Each collected strain contained at least one homologous ABG, though the gene copy numbers or types varied. This finding is consistent with a previous report, which demonstrated the broad species distribution of homologous ABGs in bacteria and fungi, contributing to diverse arsenic resistance [24]. In this study, the average copy number of bacterial homologous ABGs showed a significant positive correlation with the relative abundance of ABGs in soils, regardless of the low (*P* < 0.01) or high (*P* < 0.05) soil arsenic content, collected from the work of Wang et al. [43], as shown in Fig. S3. This indicates that the copy numbers of ABGs obtained in this study were reliable. The copy number of *arsM* was higher (*P* < 0.001) than that of the other 15 gene types. Methylation, the crucial pathway for arsenic resistance in ancient microbes [24], has now become a common pathway of arsenic metabolism in modern microbes. Furthermore, our analysis revealed that the majority of homologous *arsC1* and *arsB* genes were found in the genomes of archaea and bacteria (Fig. 1). Considering the targeted homologous ABGs among microbes, 16 gene families in bacteria, 15 in archaea (without *arsC1*), and 11 in fungi (without *arrA*, *arrB*, *arsP*, *arsD*, or *arsR*) were identified (Fig. 1). A high diversity and widespread distribution of ABGs have been observed in bacterial communities from arsenic-contaminated soils [43, 44]. Chen *et al*. discovered that 53 eukaryotic microbes lacked the homologous *arsP* and *arsR* genes [24]. ArrA and ArrB, subunits of the anaerobic respiratory reductase of As(V), are more common in facultative anaerobic bacteria [15]. In fungi, the reductases encoded by *arsC2* and *acr2* exhibit similar As(V)-reduction abilities [14]. Although fungi do not possess homologous *arsP*, they can still release gaseous arsenic, such as trivalent trimethylarsine, through polymorphic hyphae [45]. Moreover, ArsD and ArsR are As(III)-responsive repressors of the *ars* operons, which only exist in prokaryotes [46].

### Co-occurrence pattern and species distribution breadth of ABGs in prokaryotic and eukaryotic microbes

**Figure 2:**
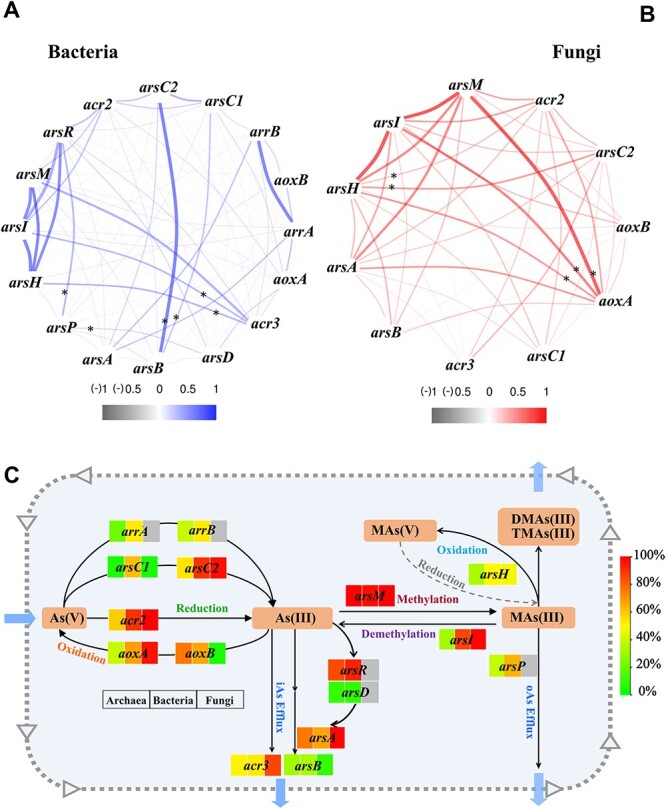
Co-occurrence pattern of ABG homologs in (**A**) prokaryotic and (**B**) eukaryotic microbes. The thickness and colour of the ribbons reflect the correlation of annotation numbers for ABGs (spearman correlation coefficient: ^*^  *P* < 0.05). (**C**) the species distribution breadth of each homologous ABG. The colour chart on the right side of the panel reflects the species distribution breadth of each ABG. “iAs” and “oAs” represent inorganic and organic arsenic, respectively.

The co-occurrence pattern of ABG homologs was investigated in prokaryotic (Fig. 2A) and eukaryotic (Fig. 2B) microbes (Fig. 2). Positive correlations (*P* < 0.001) were observed among *arsH*, *arsI*, and *arsM*, regardless of the microbe type. Notably, in prokaryotes, significant associations were found between *arsB* and *arsC2* (R = 0.538, *P* < 0.001) and *arsC1* (R = 0.339, *P* < 0.01), *acr3* and *arsI* (R = 0.294, *P* < 0.001) and *arsH* (R = 0.304, p < 0.001), and *arsP* and *arsR* (R = 0.289, *P* < 0.001) and *arsD* (R = 0.181, *P* < 0.05) (Fig. 2A). In eukaryotic microbes, significant associations were observed between *aoxA* and *arsM* (r = 0.605, *P* < 0.001), *arsI* (R = 0.457, *P* < 0.001), and *acr2* (R = 0.304, *P* < 0.001), as well as *arsH* and *aoxA* (R = 0.360, *P* < 0.001), *arsC2* (R = 0.356, *P* < 0.001), and *acr2* (R = 0.236, *P* < 0.001) (Fig. 2B). These findings indicate that in prokaryotic microbes, the efflux system mediated by *arsB*, *acr3*, and *arsP* exhibits strong associations with As(V) reduction, *ars* operator transcriptional regulation, and MAs(III) demethylation and oxidation. In eukaryotic microbes, arsenite oxidation facilitated by *aoxA* and *arsH* is strongly linked to As(V) reduction, As(III) methylation, and MAs(III) demethylation.

Among ABGs, *arsM*, *acr2*, *arsC2*, and *arsI* were widely distributed among archaea, bacteria, and fungi. For bacteria, the distribution breadth of species carrying either *acr3* or *arsB* reached 70%, which exceeds the proportion of species (52.4%) simultaneously harbouring *aoxA* and *aoxB*. The percentage of species concurrently containing both *arrA* and *arrB* reached 34.4%. Similarly, the species distribution breadth of *arsP* (64.3%) was higher than that of *arsH* (49.8%) (Fig. 2C). These results align with the findings of Chen *et al.* [24], who reported a wider distribution of the efflux genes *acr3* and *arsP* than of the oxidation gene *arsH* in bacterial genomes. The arsenic metabolism pathway in prokaryotic microbes prefers to “evict out of house” and involves the reduction of As(V) to As(III) (ArrA, ArrB, ArsC2, Acr2), followed by methylation (ArsM) to form methylarsenic (See online supplementary material for a colour version of Fig. S4). The As(III) produced is effluxed from cells through ArsB and Acr3, whereas methylarsenic is effluxed through ArsP. Prokaryotes derive energy from As(V) reduction [47]. The efflux of As(III) by Acr3 and ArsB is considered to be an economical and effective way for bacteria to detoxify arsenic [48]. Under anaerobic conditions, As(III) products can also be methylated by ArsM to produce the more hazardous MAs(III) and DMAs(III) [24]. The efflux of MAs(III) and DMAs(III) by ArsP can have an antibiotic effect, killing or suppressing certain competitors [10]. When exposed to air, these are oxidized non-enzymatically to the hypotoxic MAs(V) and DMAs(V) [24].

In fungi, the species distribution breadth of *aoxA* (95.8%) was higher than that of *acr3* (85.3%) and *arsB* (9.8%) (Fig. 2C). The species distribution breadth of *arsH* reached 47.6%, and *arsP* was not identified in fungi. The arsenic metabolism pathway in eukaryotic microbes tends to be “retention in house”. Highly toxic As(III) is readily oxidized into less toxic As(V) by enzymes encoded by *aoxA* and *aoxB*. Subsequently, the As(V) produced is not easily excreted from cells because the conversion into 1-arseno-3-phosphoglycerate (1As3PGA) is needed before efflux [49]. Some eukaryotic microbes can actively excrete As(III) through Acr3 [13] and methylate As(III) via ArsM [22]. Even in the absence of ArsP, the oxidation of MAs(III) and DMAs(III) by ArsH can produce the hypotoxic MAs(V) and DMAs(V), respectively, in cells. In addition, fungal strains release gaseous arsenic, such as trivalent trimethylarsine, through the cell wall space [50]. Many fungi also have the ability to reduce intracellular arsenic levels through biovolatilization by converting inorganic arsenic into gaseous organic forms [51]. The volatilization of gaseous arsenic helps to lower arsenic concentrations in fungal cells, thus preventing accumulation and enhancing detoxification [16].

The increase in oxygen on Earth after the Great Oxidation Event (GOE) altered arsenic speciation and geochemical cycling, thereby, intensifying the environmental pressure on arsenic metabolism by microorganisms [24]. Before the GOE, reduced arsenicals predominated owing to the anoxic and reducing conditions of the atmosphere and oceans. During this period, prokaryotes primarily engaged in As(III) and MAs(III) detoxification through processes such methylation (ArsM), efflux (Acr3 and ArsP), and reduction (ArsC2) [24]. After the GOE, the intense oxidative weathering of arsenic-bearing minerals led to the widespread appearance of oxidized arsenicals in the environment. This environmental shift drove the emergence of new arsenic pathways, such as oxidation (ArsH) and demethylation (ArsI). These drastic shifts in the redox state of arsenicals and their bioavailability imposed strong selective pressure on microorganisms to develop novel enzymatic systems for arsenic resistance [24]. The differences in arsenic metabolism pathways between prokaryotic and eukaryotic microbes are closely linked to their adaptation to oxygenation conditions and arsenic exposure. Our results suggest that the prokaryotic arsenic metabolism pathway reflects the methods that microbes used to metabolize arsenic before the GOE, primarily through reduction and efflux, whereas the eukaryotic arsenic metabolism pathway might represent the method used by microbes to metabolize arsenic after the GOE, primarily through oxidation.

**Figure 3:**
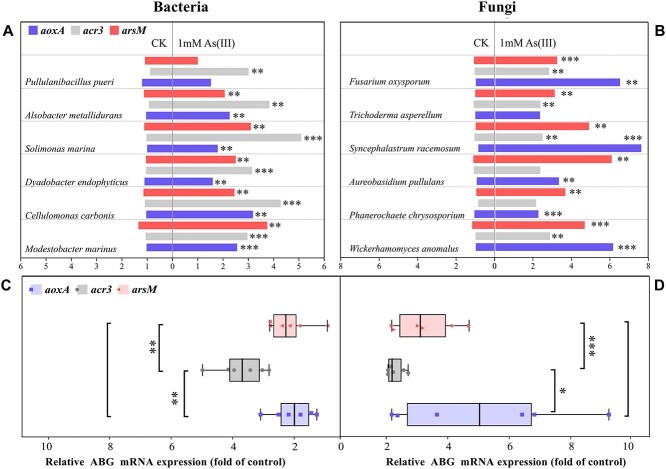
Gene expression of the key ABGs in (**A**) bacteria and (**B**) fungi, without (CK) or with 1 mM As(III). Fold-changes in gene expression in (**C**) bacteria and (**D**) fungi exposed to arsenic. Bars represent the standard error of the mean (n = 3) (Student’s *t*-test; ^*^P < 0.05, ^*^^*^P < 0.01, ^*^^*^^*^  *P* < 0.001).

To validate the key differences in arsenic metabolism between prokaryotic and eukaryotic microbes, we investigated the intracellular expression levels of *aoxA*, *acr3*, and *arsM* at the mRNA level in each of the six strains of bacteria and fungi, with or without exposure to 1 mM As(III). The expression of *aoxA*, *acr3*, and *arsM* was significantly upregulated in bacteria (*P* < 0.01; Fig. 3A) and fungi (*P* < 0.05; Fig. 3B) after As(III) exposure (Fig. 3). Compared with that in microbes without As(III) exposure, the relative expression of *aoxA*, *acr3*, and *arsM* was increased by 2.05-, 3.72-, and 2.15-fold in bacteria (Fig. 3C) and by 5.11-, 1.54-, and 4.13-fold in fungi, respectively (Fig. 3D). Notably, in bacteria, the upregulation of *acr3* expression was significantly greater than that of *arsM* (*P* < 0.005) and *aoxA* (*P* < 0.01). Conversely, in fungi, the expression of *aoxA* was higher than that of *acr3* (*P* < 0.001) or *arsM* (*P* > 0.05). This strongly supports the observation that bacteria primarily excreted As(III) using Acr3 for arsenic detoxification, whereas fungi tended to oxidize As(III) using AoxA. Similar results were observed for *Rhodococcus aetherivorans* BCP1, in which *acr3* expression was higher than *arsC2*, *arsA*, and *arsD* expression during arsenic exposure [52].

### Purifying selection of ABGs drives distinctive arsenic metabolism pathways in prokaryotic and eukaryotic microbes

**Figure 4:**
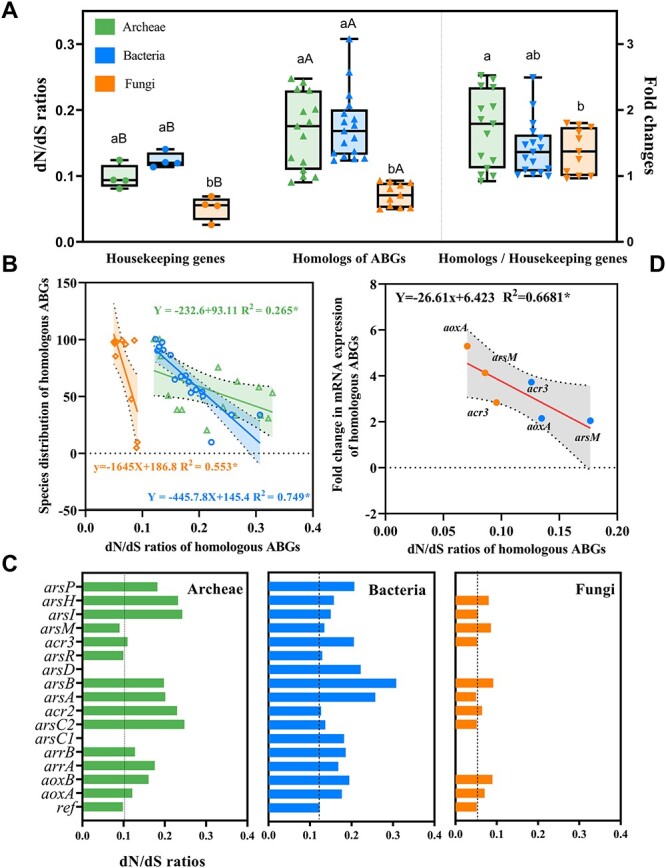
(**A**) The dN/dS values of housekeeping genes and homologous ABGs (left-side Y-axis) and fold changes in dN/dS (right-side Y-axis). Bars represent the standard error of the mean. (**B**) Correlation between dN/dS and species distribution of homologous ABGs. (**C**) dN/dS of each homologous ABG. (**D**) Correlation between the dN/dS and fold-change in homologous ABGs at the mRNA level. Different lowercase letters indicate significant differences among microbes; capital letters indicate significant differences based on a pairwise analysis. Pearson correlation coefficients: ^*^P < 0.05, ^*^^*^P < 0.01, ^*^^*^^*^  *P* < 0.001.

The dN/dS values of housekeeping genes (*recA*, *gyrB*, *fusA*, and *ileS*) and homologous ABGs in archaea, bacteria, and fungi were investigated (Fig. 4A). Those for both reference genes and homologous ABGs were all <1, indicating that the prevalence of purifying selection is the main driver of the sequence variability in ABGs. Similar studies on the housekeeping genes *recA* (dN/dS = 0.026) [53], *gyrB* (0.068) [54], and *fusA* (0.023) [55] also supported the presence of purifying selection. In this study, the average dN/dS values of housekeeping genes in archaea (0.098) and bacteria (0.124) were significantly higher (*P* < 0.05) than those in fungi (0.051). For homologous ABGs, the average dN/dS values in archaea (0.171) and bacteria (0.177) were also remarkably higher (*P* < 0.001) than those in fungi (0.073). This means that conserved genes and homologous ABGs in prokaryotic microbes exhibit higher nonsynonymous mutation rates and are more prone to amino acid-altering substitutions than those in eukaryotic microbes during evolution. A similar result was observed for *arsM* in archaea (dN/dS = 0.173) and bacteria (0.20), with a stronger nonsynonymous mutation rate than that in fungi (0.16) [23]. Interestingly, in this study, ABGs showed significantly higher (*P* < 0.05) average dN/dS values than housekeeping genes, regardless of the type of microbe. This indicates that ABGs experience a more relaxed purifying selection than housekeeping genes during evolution.

Significant negative correlations were observed between the dN/dS values of ABGs and the distribution breadth of species containing ABGs of archaea (*P* < 0.05), bacteria (*P* < 0.001), and fungi (*P* < 0.01) (Fig. 4B). This indicates that ABGs with stronger purifying selection tend to be more widely distributed across microbial species. A negative association between the species distribution breadth of a gene and the strength of purifying selection was also observed in other biological systems. For example, in multicellular organisms, the slowly evolving brain-related genes tend to have a wider distribution breadth across different tissues [35, 56]. Specifically, in bacteria, the dN/dS values of *acr2* (0.127), *arsC2* (0.137), and *acr3* (0.126) were lower than those of *aoxA* (0.177), *aoxB* (0.194), and *arsH* (0.158) (Fig. 4C). In fungi, the dN/dS values of *aoxA* (0.071), *aoxB* (0.090), and *arsH* (0.081) were lower than those of *arsB* (0.0914) and *acr3* (0.093) (Fig. 4C). This also supports the aforementioned finding that bacteria prefer the reduction and efflux of As(III), whereas fungi are involved in oxidation reactions.

We also found that the dN/dS values of ABGs were strongly and negatively associated (*P* < 0.05) with their expression at the mRNA level (Fig. 4D). This finding indicates that microbes carrying ABGs with a strong purifying selection tend to maintain their high expression levels in the environment. A similar association between gene expression and the strength of purifying selection was observed in *Saccharomyces cerevisiae* [57, 58] and mammalian genomes with high coverage [59]. The strength of purifying selection in the environment facilitates gene abundance in microbes [56]. A clear positive correlation (*P* < 0.001) between the abundance of *arsB* or *acr3* and As(III) concentrations was found by Poirel et al. [60]. Purifying selection plays a crucial role as an evolutionary pathway for microbes to maintain the essential properties of genes, such as ABGs, over long periods of time [61]. Owing to variations in the biological structure and ecological niche, particularly in relation to arsenic, ABGs in prokaryotic and eukaryotic microbes can exhibit distinct strengths and modes of selection during natural selection. These differences, in turn, influence the species distribution breadth of ABGs and gene expression level in microbes, contributing to the different arsenic metabolism pathways in eukaryotic and prokaryotic microbes and consequently affecting the environmental fate of arsenic in nature.

## Conclusions

To the best of our knowledge, this is the first research to reveal distinct arsenic metabolic pathways in prokaryotic and eukaryotic microbes. Comparatively, the arsenic metabolism pathway in prokaryotic microbes prefers to “evict out of house” and involves the reduction and efflux of arsenic. However, that in eukaryotic microbes tends to comprise “retention in house” via the oxidation of arsenic. Additionally, we demonstrated the crucial role of purifying selection in shaping the distribution and evolutionary dynamics of ABGs across different species. The conservation of these genes suggests their fundamental role in the adaptation to arsenic and underscores the significance of their expression and regulation during the microbial arsenic detoxification process. The advances reported in this study facilitate a significant change from studying individual or several ABGs to characterizing the comprehensive microbial strategy of arsenic detoxification. Understanding the interaction between arsenic and microbes also provides insights into future bio-remediation applications. The various arsenic metabolism genes offer a valuable resource, as they represent microbial strategies for controlling arsenic migration and toxicity. The molecular mechanisms underlying the purifying selection of each ABG gene is currently not understood, and additional fundamental experiments are necessary to address these issues. Moreover, the limited number of strains used in the arsenic exposure experiments also represents a limitation. Future studies should use a wider range of experimental strains and incorporate controls for environmental factors to further improve our understanding of this subject.

## Supplementary Material

Supplemental_information_ycae106

Dataset_S1_ycae106

Dataset_S2_ycae106

## Data Availability

All data generated or analysed during this study are included in this article or in the Supplementary materials.
