# Supplementary material for: Purifying selection drives distinctive arsenic metabolism pathways in prokaryotic and eukaryotic microbes
Source: ISME Commun. 2024 Aug 20;4(1):ycae106. doi: 10.1093/ismeco/ycae106 (PMC11370035; doi:10.1093/ismeco/ycae106)
Supplement: Supplemental_information_ycae106 [file supplemental_information_ycae106.docx]

**Supporting Information**

**Purifying selection drives distinctive arsenic metabolism pathways in prokaryotic and eukaryotic microbes**

*Lijuan Li^a^, Songcan Chen^b^, Ximei Xue^c^, Jieyin Chen^d^, Jian Tian^e^, Lijuan Huo^f^, Tuo Zhang^g^, Xibai Zeng^a^, Shiming Su^a^**

^a^ Institute of Environment and Sustainable Development in Agriculture, Chinese Academy of Agricultural Sciences/Key Laboratory of Agricultural Environment, MARA, Beijing 100081, P.R. China

^b^ Division of Microbial Ecology, Center for Microbiology and Environmental Systems Science, University of Vienna, Vienna 1030, Austria

^c^ Institute of Urban Environment, Key Laboratory of Urban Environment and Health, Chinese Academy of Sciences, Xiamen 361021, P.R. China

^d^ Institute of Plant Protection, State Key Laboratory for Biology of Plant Diseases and Insect Pests, Chinese Academy of Agricultural Sciences, Beijing 100193, P.R. China

^e^ Biotechnology Research Institute, Chinese Academy of Agricultural Sciences, Beijing 100081, P.R. China

^f^ School of Environment and Resources, Taiyuan University of Science and Technology, Taiyuan 030024, P.R. China

^g^ School of Environmental and Life Science, Nanning Normal University, Nanning 530100, P.R.China

Running title: Arsenic metabolism pathways in microbes

*Corresponding author: [sushiming@caas.cn](mailto:sushiming@caas.cn)

**This supporting information includes 1 Text, 4 Tables, and 4 Figures.**

**Text S1: Methods**

*1.1 Quantitative real-time PCR (qPCR)*

To verify the key pathways of arsenic metabolism and the expression of related genes in prokaryotic and eukaryotic microbes, the latter were cultured in the presence or absence of As(III). For fungi, after 2 days of incubation at 25 °C, 0.3 mL of cell suspension (10^4^ CFU·mL^-1^) was inoculated into 30 mL of medium containing 1 mM sodium arsenite (NaAsO_2_) (Sigma Chemical Co., St. Louis, MO, USA). Fungi were cultured at 25 °C and under shaking at 140 rpm, and fungal samples were harvested after 7 days, once they reached a maximum growth concentration of 2.0 < OD_600_ < 4.0 (varying with each fungal strain). For bacteria, strains were incubated in 5 mL of medium under shaking (200 rpm) at 37 °C overnight. Cells were then diluted 100-fold into 30 mL of medium containing 1 mM NaAsO_2_. Bacteria were cultured at 37 °C and under shaking at 200 rpm, and bacterial samples were harvested after 5 days, once they reached a maximum growth concentration of 1.0 < OD_600_ < 3.0 (varying for each bacterial strain). The most suitable medium composition and culture conditions differed among strains, as detailed in Supplementary Table 2. Fungi and bacteria cultivated in a medium without arsenic were used as controls. According to the results of preliminary experiments, all strains exhibited good growth in a 1 mM NaAsO_2_ medium.

RNA was extracted from the various strains using the PureLink RNA Mini kit (ThermoFisher Scientific, Waltham, MA, USA) following the manufacturer’s instructions. qPCR was conducted to assess the relative expression of the 16S rRNA, 18S rRNA, *aoxA*, *acr3*, and *arsM* genes. The primers and PCR conditions for each target gene are listed in Supplementary Table 3. qPCR was performed on an ABI 7500 Real-time Detection System (Applied Biosystems, Foster City, CA, USA) in a 20 μL mixture containing 10 μL SYBR Premix Ex Taq II (TaKaRa, Dalian, China), 2 μL cDNA template (10 ng·μL^–1^), and 0.4 μL of each primer (10 μM) (Supplementary Table 3). The thermal cycling parameters were 95 °C (10 min), followed by 40 cycles at 95 °C (15 s) and 60 °C (60 s). All samples were analysed in triplicate, and the relative expression of target genes was calculated using the 2^−ΔΔCT^ method.

*1.2 Statistical Analysi****s***

Statistical analysis was conducted by using IBM SPSS Statistics 21.0 (IBM, Endicott, NY, USA). This involved calculating the percentage of species containing homologous ABGs, means, statistical significance (parametric and nonparametric tests), standard deviation (SD), and conducting simple linear regression analysis. For data visualization, Origin Pro 2021 (OriginLab, Northampton, MA, USA) and GraphPad Prism 9 (GraphPad Software Inc., San Diego, CA, USA) were used to generate bar graphs, boxplots, and linear regression plots. Heatmaps and co-occurrence networks were constructed using TBtools (South China Agricultural University, Guanzhou, Guangdong, China). Other graphs were plotted using Inkscape 1.2 (Software Freedom Conservancy, Brooklyn, NY, USA).

**Table S1.** Biochemical properties of 16 arsenic functional genes

| **Gene** | **Functional**  **process** | **Encoded protein** | **Domain** | **Pfam id** | **COG id** |
| --- | --- | --- | --- | --- | --- |
| ***aoxA*** | As(III) oxidation | As(III) oxidase small subunit | Rieske | PF00355 | COG0723 [1] |
| ***aoxB*** |  | As(III) oxidase large subunit | Molydop_binding Molybdopterin | PF00384  PF01568 | [1] |
| ***arrA*** | As(V) reduction | As(V) respiratory reductase large subunit | Molydop_binding Molybdopterin Molybdop_Fe4S4 | PF04879  PF00384  PF01568 | COG0243 [2] |
| ***arrB*** |  | As(V) respiratory reductase small subunit | Fer4_11 | PF13247 | COG0437 [2] |
| ***arsC1*** |  | As(V) reductase (glutaredoxin family) | ArsC | PF03960 | COG1393 |
| ***arsC2*** |  | As(V) reductase  (low-molecular-weight phosphatases family) | LMWPc | PF01451 | COG0394 |
| ***acr2*** |  | As(V) reductase  (CDC25 family of dual-specific phosphatases) | Rhodanese | PF00581 | COG0607 [3, 4] |
| ***arsR*** |  | Transcriptional repressor | HTH_20 | PF12840 | COG0640 [3] |
| ***arsI*** | Arsenic demethylation | C-As lyase | Glyoxalase | PF00903 | COG0346 [5] |
| ***arsM*** | Arsenic methylation | ArseniteS-adenosylmethionine (SAM) methyltransferase | Methyltransfer_11 | PF08241 | COG2226 [6] |
| ***arsA*** | As(III)  efflux | As(III)-activated ATPase | ArsA_ATPase | PF02374 | COG0003 [7] |
| ***arsB*** |  | As(III) efflux pump protein | ArsB | PF02040 | COG1055 [8] |
| ***arsD*** |  | Arsenical metallochaperone | ArsD | PF06953 | [9] |
| ***acr3*** |  | As(III) efflux pump protein | SBF | PF01758 | COG0798 [4] |
| ***arsP*** | Trivalent organoarsenical  permease | Trivalent organoarsenical permease | ArsP | PF03773 | COG0701 [10] |
| ***arsH*** | Organoarsenical  oxidation | Organoarsenical oxidase | FMN_red | PF03358 | COG0431 [11] |

Note: Pfam, protein domain database; COG, Clusters of homologous Group.

**Table S2.** Source and culture information for the 12 strains used in this study

| **Species name** | **GenBank or JGI id** | **Strain number** | **Medium used** |
| --- | --- | --- | --- |
| *Aureobasidium pullulans* | AurpulNBB1 | cgmcc 3.2756 | 0014 PDA medium |
| *Syncephalastrum racemosum* | Synrac1 | cgmcc 3.3464 | 0017 Comprehensive PDA |
| *Wickerhamomyces anomalus* | Wican1 | cgmcc 2.4313 | 0013 Wort medium |
| *Phanerochaete chrysosporium* | Phchr2 | [cgmcc 3.7212](https://cgmcc.net/directory/detail?cgmccid=3.7212&number=&genus=&species=&yiming=%E9%BB%84%E5%AD%A2%E5%8E%9F%E6%AF%9B%E5%B9%B3%E9%9D%A9%E8%8F%8C&page=1) | 0091 Potato medium |
| *Fusarium oxysporum* | FoxFo5176 | cgmcc 3187 | 0014 PDA medium |
| *Trichoderma asperellum* | GCA_014839745.1 | cgmcc 3187 | 0014 PDA medium |
| *Modestobacter marinus* | GCF_014645755.1 | cgmcc 4.5581 | 0038 ISP-2 medium |
| *Cellulomonas carbonis* | GCF_014636275.1 | [cgmcc 1.10786](https://cgmcc.net/directory/detail?cgmccid=1.10786&number=&genus=&species=&yiming=%E7%85%A4%E7%BA%A4%E7%BB%B4%E5%8D%95%E8%83%9E%E8%8F%8C&page=1) | 0908 R2A medium |
| *Alsobacter metallidurans* | GCF_014636935.1 | [cgmcc 1.12214](https://cgmcc.net/directory/detail?cgmccid=1.12214&number=&genus=&species=&yiming=%E8%80%90%E9%87%91%E5%B1%9E%E6%9E%9C%E5%9B%AD%E6%9D%86%E8%8F%8C&page=1) | 0002 Nutrient gravy medium |
| *Dyadobacter endophyticus* | GCF_014641595.1 | [cgmcc 1.15288](https://cgmcc.net/directory/detail?cgmccid=1.15288&number=&genus=&species=&yiming=%E6%A4%8D%E7%89%A9%E5%86%85%E7%94%9F%E6%88%90%E5%AF%B9%E6%9D%86%E8%8F%8C&page=1) | 0033 LB medium |
| *Solimonas terrae* | GCF_012241385.1 | cgmcc 1.16161 | 0904 R2A medium |
| *Pullulanibacillus pueri* | GCF_014639255.1 | cgmcc 1.12777 | 0002 Nutrient gravy medium |

Details of strain characteristics and culture are available at https://www.cgmcc.net/.

JGI, Joint Genome Institute

**Table S3.** Primer information for genes in bacterial strains analysed in this study

| **Strain** | **Primer** | **Sequence** |
| --- | --- | --- |
| *Modestobacter marinus* | *aoxA*-1F | 5′-CTACGACGGCGTGGAGGT-3′ |
|  | *aoxA*-1R | 3′-GAGGAGCGGGGTGAAGAA-5′ |
|  | *acr3*-1F | 5′-CTCGGTGTTCCAAATCCT-3′ |
|  | *acr3*-1R | 3′-TACCAGTCCCGTCCCTTG-5′ |
|  | *arsM*-1F | 5′-CGGCGTGGTCATCTCCTT-3′ |
|  | *arsM*-1R | 3′-CCGCATCAGGTACTCGGT-5′ |
| *Cellulomonas carbonis* | *aoxA*-2F | 5′-TCAAGGCATCCGAGGTCA-3′ |
|  | *aoxA*-2R | 3′-ACAGCAGGTGGTGCGTCT-5′ |
|  | *acr3*-2F | 5′-TCGGTGCTCGGCTGGTTC-3′ |
|  | *acr3*-2R | 3′-CGTGAAGAGCAGCCCGTA-5′ |
|  | *arsM*-2F | 5′-TCGTCCCCTGCGACCTGT-3′ |
|  | *arsM*-2R | 3′-GCGTCACGCGGAGCATCT-5′ |
| *Dyadobacter endophyticus* | *aoxA*-3F | 5′-ACAAAGCTGAAAACCAACGG-3′ |
|  | *aoxA*-3R | 3′-GGCAATGCTGAATGTGGA-5′ |
|  | *acr3*-3F | 5′-TCAGCCCGATCACCTTAA-3′ |
|  | *acr3*-3R | 3′-GTAGATTTGGCATAGTCCG-5′ |
|  | *arsM*-3F | 5′-CCGTATTGTCGGACCTTT-3′ |
|  | *arsM*-3R | 3′-CATCACCTACCACCACTTTT-5′ |
| *Solimonas marina* | *aoxA*-4F | 5′-CCGATTCCTGACGCTGAC-3′ |
|  | *aoxA*-4R | 3′-GGTTGGACTTCGCTGATG-5′ |
|  | *acr3*-4F | 5′-TCGCTACCTGACGCTGTG-3′ |
|  | *acr3*-4R | 3′-GGATGCGGCTGTCCTTGA-5′ |
|  | *arsM*-4F | 5′-CCGCCGACTACACCCACT-3′ |
|  | *arsM*-4R | 3′-CGAAACGCTTCCAGACCC-5′ |
| *Alsobacter metallidurans* | *aoxA*-5F | 5′-AGCTGATCTTCGTCCGTC-3′ |
|  | *aoxA*-5R | 3′-CGTACACCACGAGCCACT-5′ |
|  | *acr3*-5F | 5′-ACGCTATGGGTGGGCTTGT-3′ |
|  | *acr3*-5R | 3′-CTGGCAGGTTCACTTTCG-5′ |
|  | *arsM*-5F | 5′-CGATGGTGGACGAGGTGT-3′ |
|  | *arsM*-5R | 3′-ACGCAGGGTGGAGACGAG-5′ |
| *Pullulanibacillus pueri* | *aoxA*-6F | 5′-ACCACAGTCCCCTGAACC-3′ |
|  | *aoxA*-6R | 3′-CCCGTAATCCCACCACCT-5′ |
|  | *acr3*-6F | 5′-AGCACCCATCTTAACCCC-3′ |
|  | *acr3*-6R | 3′-GAGAATCAGGCGAACAACAA-5′ |
|  | *arsM*-6R | 5′-TGCAGGAGAATGGGAAAC-3′ |
|  | *arsM*-6F | 3′-CCTACAACAGAAGACGCT-5′ |
| Bacteria | 16S rRNA-F | 5′-CCTACGGGAGGCAGCAG-3′ |
|  | 16S rRNA-R | 3′-ATTACCGCGGCTGCTGG-5′ |

| **Strain** | **Primer** | **Sequence** |
| --- | --- | --- |
| *Wickerhamomyces anomalus NRRL*  *Y-366-8* | *aoxA*-7R | 5′-CACAATGTCTGCCTCTGC-3′ |
|  | *aoxA*-7R | 3′-GGTTTTGAACACGGTCTG-5′ |
|  | *acr3*-7F | 5′-GGTGGTGCTCTGCCTCAT-3′ |
|  | *acr3*-7R | 3′-GCAGCTATGGCCTGTCGA-5′ |
|  | *arsM*-7F | 5′-TACCAGTTTTCGGTCAAGC-3′ |
|  | *arsM*-7R | 3′-GCGACACCAAATGTCAAG-5′ |
| *Phanerochaete chrysosporium* | *aoxA*-8F | 5′-TGCGAAGAAGCCTGAGTG-3′ |
|  | *aoxA*-8R | 3′-TGTCGTAGTGGGAACCGT-5′ |
|  | *acr3*-8R | 5′-GGTCGTCCAGGCGTTCAC-3′ |
|  | *acr3*-8F | 3′-GCAGCCCCGTCTTCTCAT-5′ |
|  | *arsM*-8F | 5′-TACCTCATCCCGCACCTC-3′ |
|  | *arsM*-8R | 3′-ATGTCGAACGACCCGTCT-5′ |
| *Aureobasidium*  *Pullulans* | *aoxA*-9F | 5′-ACTTCAAGAAATACCGCAGCAC-3′ |
|  | *aoxA*-9R | 3′-CGGCAAGATCAATCTCAACC-5′ |
|  | *acr3*-9F | 5′-AAGATTTCACCGACCTGG-3′ |
|  | *acr3*-9R | 3′-TGGCGAAGAAGTAGACGA-5′ |
|  | *arsM*-9F | 5′-ATGTCTTCGTTCGTTCCTC-3′ |
|  | *arsM*-9R | 3′-CACCGACCTTTGACTCCT-5′ |
| *Syncephalastrum racemosum* | *aoxA*-10F | 5′-CTGCTATGGCTAAGGTCC-3′ |
|  | *aoxA*-10R | 3′-CTAATGGGGTTGGTGTTG-5′ |
|  | *acr3*-10F | 5′-GTGACCCAGGCATTTACG-3′ |
|  | *acr3*-10R | 3′-AGCAACTTCTTTGGAGGC-5′ |
|  | *arsM*-10F | 5′-CTCCCTCGCCTTCCATTA-3′ |
|  | *arsM*-10R | 3′-CGCCTTGTCGTTGTCTCC-5′ |
| *Trichoderma asperellum* | *aoxA*-11F | 5′-AACTGCTTCCCGCCTGTG-3′ |
|  | *aoxA*-11R | 3′-CTCCCATTGTGCCGACCA-5′ |
|  | *acr3*-11F | 5′-CGGAGGCGACAACGAATA-3′ |
|  | *acr3*-11R | 3′-CCCAAGGAACACTGCGAC-5′ |
|  | *arsM*-11F | 5′-GGCTGCCACAGACGACAT-3′ |
|  | *arsM*-11R | 3′-GCCATTGAGGCCAAGATC-5′ |
| *Fusarium oxysporum* | *aoxA*-12F | 5′-ACACCGAACCCAGGACGA-3′ |
|  | *aoxA*-12R | 3′-GCAAGGGCAGAACCAACC-5′ |
|  | *acr3*-12F | 5′-TGGGCTTTTCTCCCTGAC-3′ |
|  | *acr3*-12R | 3′-ACCCGACTCGTGACTGATG-5′ |
|  | *arsM*-12F | 5′-CCCCAATGTCTCCGTTAT-3′ |
|  | *arsM*-12R | 3′-GAGCGAGTTATCCTCAATCT-5′ |
| Fungi | 18S rRNA-F | 5′-TGTTCAGCAGCATCGTCTCG-3′ |
|  | 18S rRNA-R | 3′-TGCCAAAGTCGCTCGGGTAG-5′ |

**Table S4.** Primer information for genes in fungal strains analysed in this study

**Table S5.** Information of housekeeping genes analysed in this study

| **Gene name** | **Function** | **NCBI HMM accession** | **Reference** |
| --- | --- | --- | --- |
| *recA* | DNA strand exchange and recombination protein | TIGR02012.1 | TIGR02012.1 [12] |
| *gyrB* | DNA gyrase subunit B | TIGR01059.1 | TIGR01059.1 [13] |
| *fusA* | GTP-binding protein chain elongation factor EF-G | TIGR00484.1 | TIGR00484.1 [14] |
| *iles* | Isoleucine tRNA synthetase | TIGR00392.1 | TIGR00392.1 [15] |

NCBI, National Center for Biotechnology Information**;** HMM, hidden Markov mode





Figure S1**.** Phylogenetic relationship analysis. A phylogenetic tree based on the proteome comparative analysis of 670 microbes was prepared using the CVTree3 software


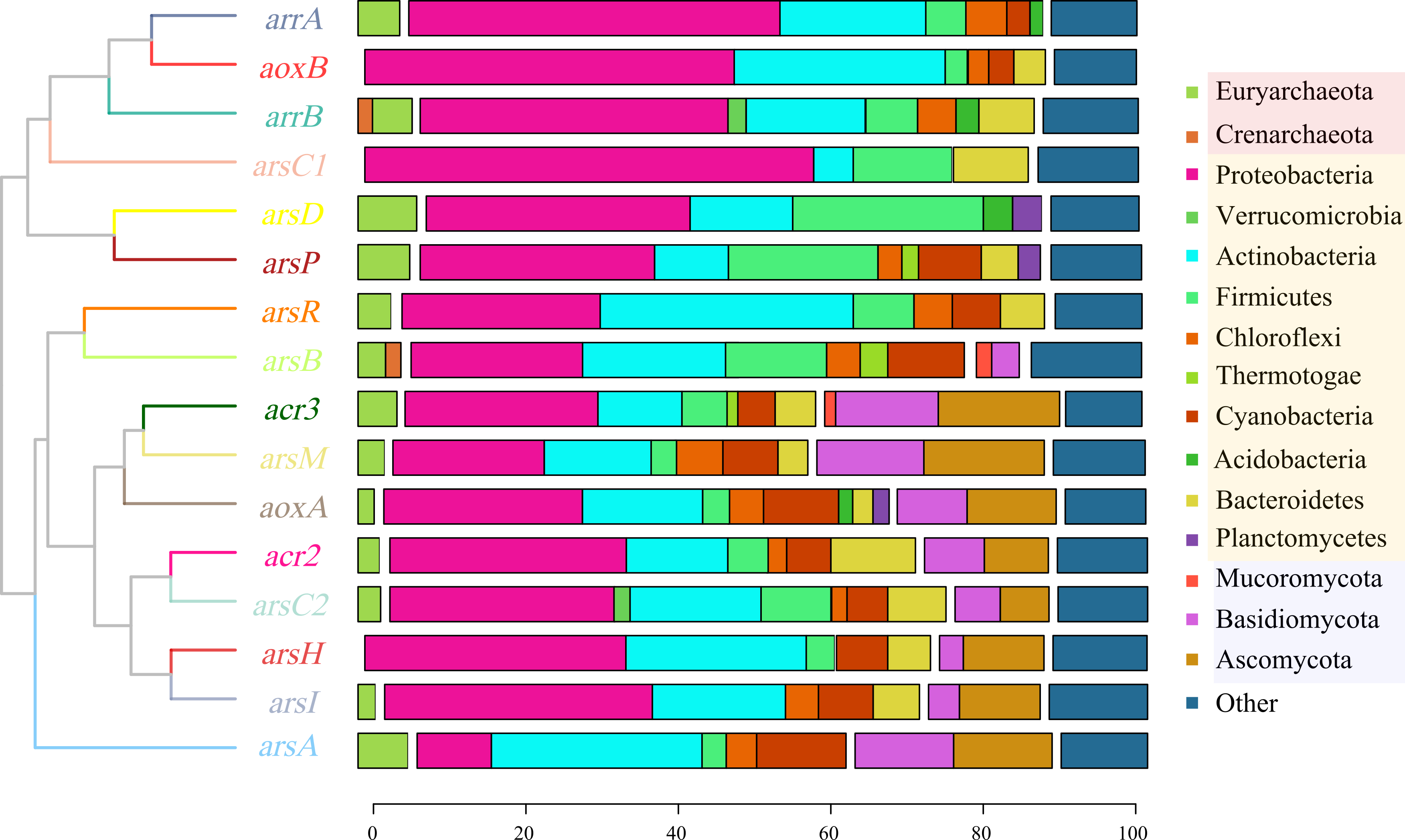


Figure S2. Species composition analysis. Differences in species composition of arsenic biotransformation genes (ABGs) at phylum-level


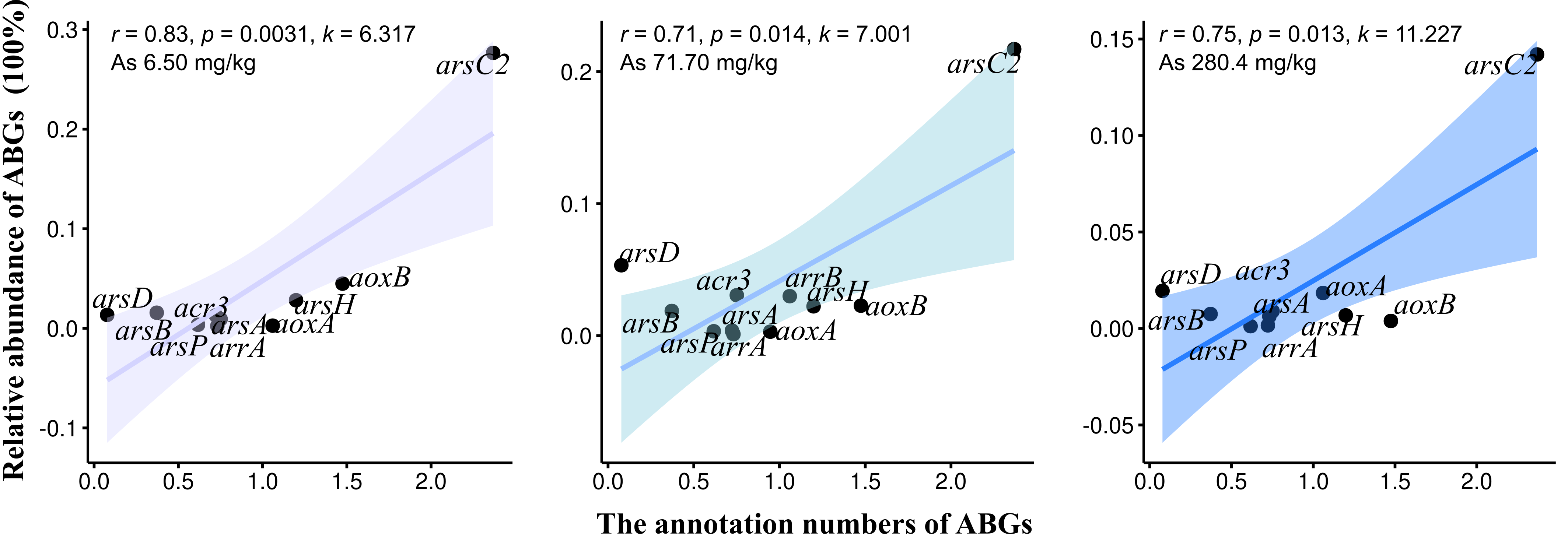


Figure S3. Linear fit of relative abundance and annotation numbers of bacterial ABGs. Linear fit of relative abundance of bacterial ABGs in different soil arsenic contents (6.5, 71.70, and 280.4 mg·kg^-1^) and annotation numbers of bacterial ABGs from this study. The ABG relative abundance data used in this linear fit were obtained from published research [16] .


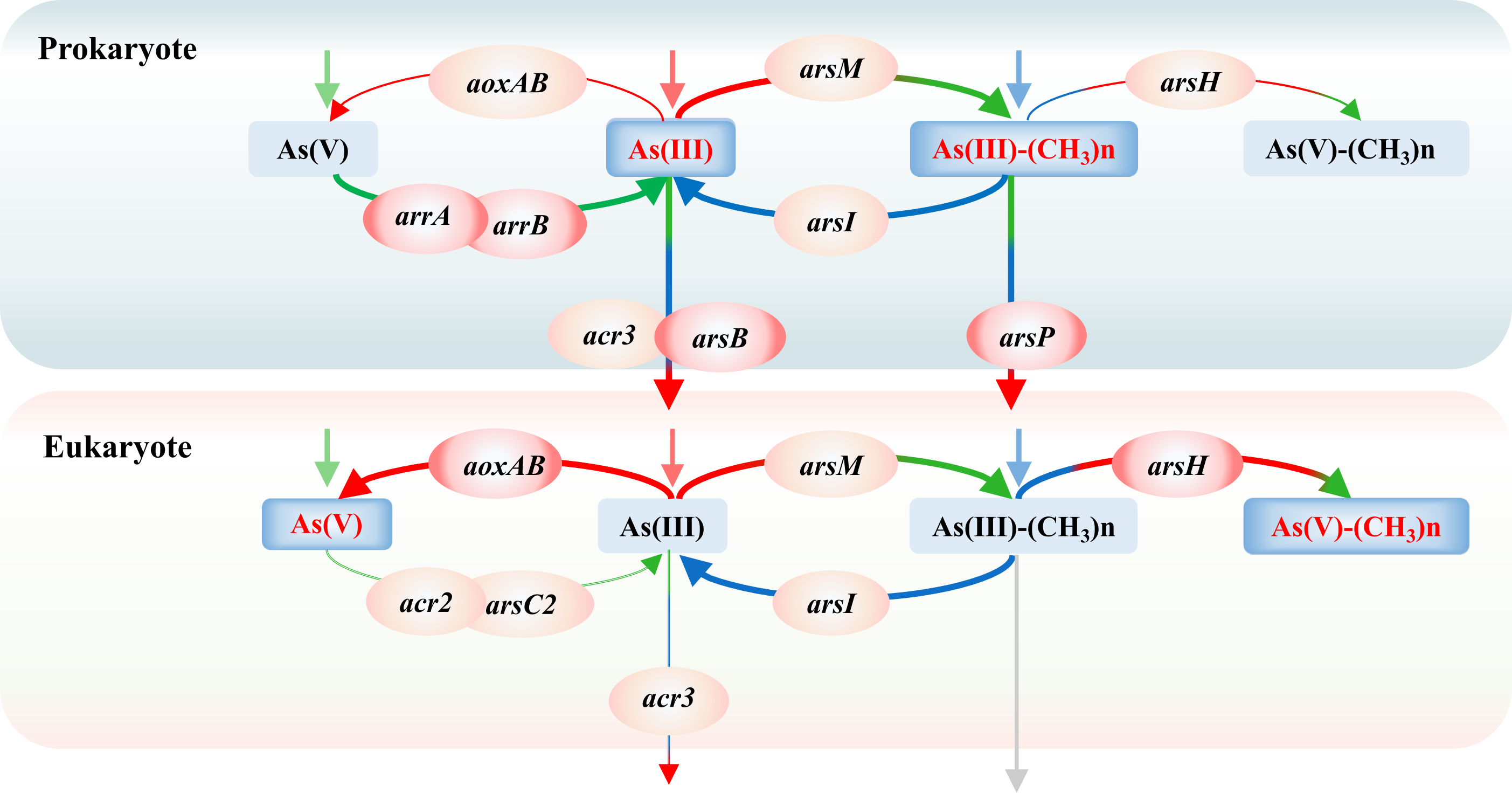


Figure S4. Arsenic metabolism pathways in prokaryotic and eukaryotic microbes

As(III)–(CH_3_)n and As(V)–(CH_3_)n represent trivalent methylated arsenic and pentavalent methylated arsenic, respectively. (1) When As(III) is the dominant species in the environment, prokaryotes often methylate As(III) to MAs(III), and then extrude it from the cell using ArsP. Only a small concentration of As(III) is oxidised by AoxAB. Conversely, in eukaryotes, some As(III) is oxidised to As(V) by AoxAB, and the remainder is methylated to MAs(III) by ArsM and then oxidised to MAs(V) by ArsH. Eukaryotic microorganisms usually transform high-toxicity As(III) and As(III)–(CH_3_)n to low-toxicity As(V) and As(V)–(CH_3_)n, respectively. (2) When As(V) is the dominant species, prokaryotes usually reduce As(V) to As(III) using various reductases (ArsC2, Acr2, ArrAB), extrude As(III) from the cell using ArsB or Acr3, or methylate As(III) to MAs(III), and then extrude MAs(III) from the cell. However, in eukaryotes, reduced As(III) efflux is not the main transformation pathway. (3) When MAs(III) is the dominant species, prokaryotes demethylate it to As(III), and then extrude As(III) or actively extrude MAs(III) using ArsP because MAs(III) is regarded as an antibiotic for bacteria to gain competitive advantage. Eukaryotic microbes always demethylate MAs(III) to As(III), and then transfer As(III) to vacuoles using Ycf1p or directly oxidise MAs(III) to MAs(V).

**SI References**

1. Muller D, Lievremont D, Simeonova DD, Hubert JC, Lett MC.. Arsenite oxidase aox genes from a metal-resistant β-proteobacterium. J Bacteriol 2003;185(1):135-141 .
2. Saltikov CW, Newman DK. Genetic identification of a respiratory arsenate reductase. Proc Natl Acad Sci 2003;100:10983-10988.
3. Chen SC, Sun GX, Yan Y, Konstantinidis KT, Zhang SY, Deng Y, et al. The Great Oxidation Event expanded the genetic repertoire of arsenic metabolism and cycling. Proc Natl Acad Sci 2020;117:10414-10421.
4. Bobrowicz P, Wysocki R, Owsianik G, Goffeau A, Ułaszewski S. Isolation of three contiguous genes, ACR1, ACR2, and ACR3, involved in resistance to arsenic compounds in the yeast *Saccharomyces cerevisiae*. Yeast 1997;13:819-828 .
5. Yan Y, Ye J, Xue XM, Zhu YG. Arsenic demethylation by a C·As lyase in *cyanobacterium Nostoc* sp. PCC 7120. Environ Sci Technol 2015;49:14350-14358.
6. Qin J, Rosen BP, Zhang Y, Wang G, Franke S, Rensing C. Arsenic detoxification and evolution of trimethylarsine gas by a microbial arsenite S-adenosylmethionine methyltransferase. Proc Natl Acad Sci 2006;103(7):2075-2080.
7. Lin Y, Walmsley AR, Rosen BP. An arsenic metallochaperone for an arsenic detoxification pump. Proc Natl Acad Sci 2006;103(42):15617-15622 .
8. Meng Y, Liu Z, Rosen BP. As (III) and Sb (III) uptake by GlpF and efflux by ArsB in *Escherichia coli*. J Biol Chem 2004;279:18334-18341.
9. Yang J, Rawat S, Stemmler TL, Rosen BP. Arsenic binding and transfer by the ArsD As (III) metallochaperone. Biochemistry 2010;49:3658-3666.
10. Chen J, Madegowda M, Bhattacharjee H, Rosen BP. ArsP: a methylarsenite efflux permease. Mol Microbiol 2015;98:625-635.
11. Chen J, Bhattacharjee H, Rosen BP. ArsH is an organoarsenical oxidase that confers resistance to trivalent forms of the herbicide monosodium methylarsenate and the poultry growth promoter roxarsone. Mol Microbiol 2015;96:1042-1052.
12. Patil KN, Singh P, Harsha S, Muniyappa K. *Mycobacterium leprae* RecA is structurally analogous but functionally distinct from *Mycobacterium tuberculosis* RecA protein. Biochim Biophys Acta - Proteins Proteomics. 2011;1814(12):1802-1811.
13. Reece RJ, Maxwell A. DNA gyrase: structure and function. Crit Rev Biochem Mol Biol Evol 1991;26:335-375.
14. Peske F, Savelsbergh A, Katunin VI, Rodnina, MV, Wintermeyer W. Conformational changes of the small ribosomal subunit during elongation factor G-dependent tRNA–mRNA translocation. J Mol Biol 2004;343:1183-1194.
15. Nakama T, Nureki O, Yokoyama S. Structural basis for the recognition of isoleucyl-adenylate and an antibiotic, mupirocin, by isoleucyl-tRNA synthetase. J Biol Chem 2001;276:47387-47393.
16. Zhao Y, Su JQ, Ye J, Rensing C, Tardif S, Zhu YG, Brandt KK. AsChip: a high-throughput qPCR chip for comprehensive profiling of genes linked to microbial cycling of arsenic. Environ Sci Technol 2018;53:798-807.
